# Using the Chinese version of Memorial Delirium Assessment Scale to describe postoperative delirium after hip surgery

**DOI:** 10.3389/fnagi.2014.00297

**Published:** 2014-11-05

**Authors:** Zhongyong Shi, Yujie Wu, Cheng Li, Shukun Fu, Guodong Li, Yingbo Zhu, Celeste A. Swain, Edward R. Marcantonio, Zhongcong Xie, Yuan Shen

**Affiliations:** ^1^Department of Psychiatry, Tenth People’s Hospital of Tongji UniversityShanghai, China; ^2^Department of Anesthesiology, Tenth People’s Hospital of Tongji UniversityShanghai, China; ^3^Department of Orthopedic Surgery, Tenth People’s Hospital of Tongji UniversityShanghai, China; ^4^School of Medicine, Tongji UniversityShanghai, China; ^5^Geriatric Anesthesia Research Unit, Department of Anesthesia, Critical Care and Pain Medicine, Massachusetts General Hospital and Harvard Medical SchoolCharlestown, MA, USA; ^6^Divisions of General Medicine and Primary Care and Gerontology, Department of Medicine, Beth Israel Deaconess Medical Center and Harvard Medical SchoolBoston, MA, USA

**Keywords:** memorial delirium assessment scale, confusion assessment method, postoperative delirium, hip surgery, Chinese

## Abstract

**Objective**: Memorial Delirium Assessment Scale (MDAS) assesses severity of delirium. However, whether the MDAS can be used in a Chinese population is unknown. Moreover, the optimal postoperative MDAS cutoff point for describing postoperative delirium in Chinese remains largely to be determined. We therefore performed a pilot study to validate MDAS in the Chinese language and to determine the optimal postoperative MDAS cutoff point for delirium.

**Methods**: Eighty-two patients (80 ± 6 years, 21.9% male), who had hip surgery under general anesthesia, were enrolled. The Confusion Assessment Method (CAM) and Mini-Mental State Examination (MMSE) were administered to the patients before surgery. The CAM and MDAS were performed on the patients on the first, second and fourth postoperative days. The reliability and validity of the MDAS were determined. A receiver operating characteristic (ROC) curve was used to determine the optimal Chinese version MDAS cutoff point for the identification of delirium.

**Results**: The Chinese version of the MDAS had satisfactory internal consistency (*α* = 0.910). ROC analysis obtained an average optimal MDAS cutoff point of 7.5 in describing the CAM-defined postoperative delirium, with an area under the ROC of 0.990 (95% CI 0.977–1.000, *P* < 0.001).

**Conclusions**: The Chinese version of the MDAS had good reliability and validity. The patients whose postoperative Chinese version MDAS cutoff point score was 7.5 would likely have postoperative delirium. These results have established a system for a larger scale study in the future.

## Introduction

Delirium is a disturbance of consciousness with an acute onset and a fluctuating nature, that is accompanied by changes in cognition or perceptual disturbances which are not attributable to pre-existing psychiatric disorders or substance-induced states (American-Psychiatric-Association, 1997). It has been suggested that surgery and anesthesia are associated with post-operative cognitive disorders including delirium (Kapila et al., 2014). It is estimated that delirium occurs in 14% to 56% of elderly patients following surgery under anesthesia, and postoperative delirium is one of the most common postoperative complications in older adults (Breitbart et al., 1997; DeCrane et al., 2011; Rudolph and Marcantonio, 2011; Marcantonio, 2012).

Postoperative delirium usually manifests itself as disorientation, cognitive impairment and alteration of mental processes; it can present itself either in a hyperactive form, a hypoactive form or a combination of these two forms (Field and Wall, 2013). Postoperative delirium has been reported to be associated with prolonged hospitalization, long-term cognitive impairment, functional deficits, increased morbidity and mortality, as well as adding to the burdens of caregivers (Meagher et al., 2000; Leslie and Inouye, 2011; Saczynski et al., 2012). Although it significantly impacts patient recovery after surgery, postoperative delirium often goes unrecognized (Neufeld and Thomas, 2013). Hence, an effective assessment, using validated tools, is important for the purpose of identifying the severity and the overlooked incidences of postoperative delirium.

The Confusion Assessment Method (CAM; Inouye et al., 1990), which has been translated into Chinese (Leung et al., 2008), is widely used to determine the prevalence of delirium. The Memorial Delirium Assessment Scale (MDAS) has been used to assess the severity of delirium based on 10 features (Breitbart et al., 1997; Marcantonio et al., 2002).

The MDAS has been translated into multiple languages, utilized in different countries, and has good reliability and validity (Grassi et al., 2001; Matsuoka et al., 2001; Shyamsundar et al., 2009; Noguera et al., 2014). However, the MDAS has not been translated into Chinese, and it remains unknown whether it can be used to identify postoperative delirium and assess its severity in Chinese people. Therefore, we set out to perform a prospective investigation with 82 Chinese patients, who had hip surgery under general anesthesia, in Shanghai, P.R. China, to assess the validity and reliability of MDAS in a Chinese population. Moreover, we determined the optimal MDAS cutoff point for describing the postoperative delirium of the Chinese patients, defined by CAM. The primary objective was to determine whether the MDAS had good reliability and validity in the Chinese language. The secondary objective was to assess the extent to which there was an optimal postoperative cutoff point in the Chinese version of MDAS; the scores above this cutoff point would be strongly associated with the presence of delirium, as determined by the CAM diagnostic algorithm.

## Methods

### Participants

The protocol was approved by the Human Research Ethics Committee of the Tenth People’s Hospital in Shanghai, P. R. China [RES- 2013015]. A total of 130 patients, who had hip fractures and were admitted to the Department of Orthopedics in the Tenth People’s Hospital, were screened and asked to participate in the study. Participants were included if they met the following eligibility criteria: (1) age 65 years or older; (2) patients who had hip replacements or open reductions with internal fixation (ORIF) under general anesthesia for the repair of hip fractures. Patients were excluded from participation if they had: (1) cognitive impairment at enrollment (MMSE scores less than 18); and/or (2) pre-existing delirium, cerebrovascular disorders or mental disorders (e.g., depression or schizophrenia), diagnosed by using the Diagnostic and Statistical Manual of Mental Disorders (DSM-IV) (American-Psychiatric-Association, 1997). All participants signed the written informed consent before being enrolled in the study. The participants were screened for the study from August, 2013 to December, 2013. One hundred and thirty participants were enrolled and the data from 82 participants were included for the final data analysis (see the Flow diagram). Sample size was calculated by determining the difference in MDAS scores between the participants with delirium and the participants without delirium in our pilot study with 80% power and 5% type I error.

#### Pre-operative interview

Screening assessments were performed 1 day before the scheduled surgery and included demographic characteristics (e.g., age, gender and education), medical information (e.g., diagnosis and type of surgery), and evaluation of cognitive function using the Mini-Mental State Examination (MMSE). The CAM was also performed on the participants one day before the surgery.

#### Anesthesia and surgery

All of the participants had hip replacements or open reductions with internal fixation under general anesthesia for the repair of hip fractures. The participants had standardized perioperative care, including preoperative medication, general anesthesia, and postoperative pain control. The participants were given midazolam (1.6 ± 0.59 mg, intravenous administration) as preoperative medication. The general anesthesia was induced by intravenous administration of propofol (95.56 ± 42.51 mg), sufentanil (14.45 ± 6.51 µg), and cisatracurium (10.52 ± 4.51 mg). The general anesthesia was maintained by using propofol (295.66 ± 121.14 mg), remifentanil (0.89 ± 0.28 mg), sevoflurane (21.54 ± 6.78 ml), and cisatracurium (7.52 ± 3.45 mg). The postoperative pain control included a standard postoperative pain management, e.g., sufentanil and butorphanol patient-controlled analgesia (0.5 µg sufentanil and 0.0125 mg butorphanol per injection, interval time of injection was 15 min with a total of 2 µg sufentanil and 0.05 mg butorphanol per hour). There were no major complications among the participants during the immediate postoperative period.

#### Post-operative interview

The assessment of delirium was performed after surgery once per day between 8:00 am and 2:00 pm. Patient charts were not reviewed for episodes of delirium, which could have occurred outside the time of assessment. The prevalence of postoperative delirium was assessed by a psychiatrist, (Yujie Wu), according to the CAM diagnostic algorithm. The severity of delirium was determined with the MDAS by another psychiatrist, (Zhongyong Shi), who was blinded to the results of the CAM. The psychiatrists who performed the delirium assessments in the current study had good training and went through quality control procedures. In the current study, the CAM and MDAS were conducted on the first (day 1), second (day 2) and fourth (day 4) day after the surgery by these psychiatrists, because postoperative delirium occurs most often on postoperative day 1 and 2. We performed the CAM and MDAS on postoperative day 4 to detect late-occurring postoperative delirium cases.

##### Confusion Assessment Method (CAM)

The CAM algorithm consists of four clinical criteria: (1) acute onset and fluctuating course; (2) inattention; (3) disorganized thinking; and (4) altered level of consciousness. For delirium to be defined, both the first and the second criteria have to be present, plus either: the third or the fourth criteria present, or both the third and forth criteria present together (Inouye et al., 1990). The CAM in the Chinese language has been proven to have good reliability and validity with use in the Chinese elderly population (Leung et al., 2008).

##### Memorial Delirium Assessment Scale (MDAS)

The original MDAS is designed to assess the severity of delirium symptoms, and it contains ten items: (1) awareness; (2) orientation; (3) short-term memory; (4) digit span; (5) attention capacity; (6) organizational thinking; (7) perceptual disturbance; (8) delusions; (9) psychomotor activity; and (10) sleep-wake cycle (Breitbart et al., 1997). Each item is rated from 0 (none) to 3 (severe) depending on the level of impairment. Translation and back-translation methods were used to create the Chinese version of the MDAS. The MDAS was first translated into Chinese by Yingbo Zhu and then back-translated into English by Zhongyong Shi. The original English version and the back-translated English version of the MDAS were compared, and the translation variations were inspected for consistency. All of the items with variances were then translated into Chinese and back-translated into English again according to suggestions from consistency discussions. The final Chinese version of the MDAS was generated only if its back-translated English version was consistent with the original English version of the MDAS.

#### Statistics

Participants’ characteristics, including age, height, weight, education, length of anesthesia, length of operation, estimation of blood loss and MDAS and MMSE scores, were presented as means ± standard deviation (SD). Continuous parameters were compared with the Analysis of Variance (ANOVA) or Student *t*-test. Categorical factors, such as gender, were compared with the Chi-square test. All analyses were performed using SPSS version 20.0 (SPSS Inc., Chicago, IL) with *P* < 0.05 as the significance level.

##### Reliability

Reliability was determined by using Cronbach’s alpha coefficient. The Cronbach’s alpha coefficient was calculated to assess internal consistency between MDAS items (inter-item reliability).

##### Validity

Concurrent validity of the MDAS was evaluated by Student *t*-test, comparing the MDAS scores (average of day 1, 2 and 4 after surgery) between patients with or without delirium, as determined by the CAM. The hypothesis was that higher MDAS scores would be associated with the presence of CAM-defined delirium.

##### Determination of optimal MDAS cutoff point

A receiver operating characteristic (ROC) curve was used to determine the optimal MDAS cutoff point for description of CAM-defined postoperative delirium. The total area under the curve (AUC), its 95 % confidence interval (CI), the total accuracy and the Kappa value, were all used for this determination. The optimal MDAS score was calculated as: (maximum of [sensitivity + specificity − 1]).

## Results

### Characteristics of participants

**Figure 1:**
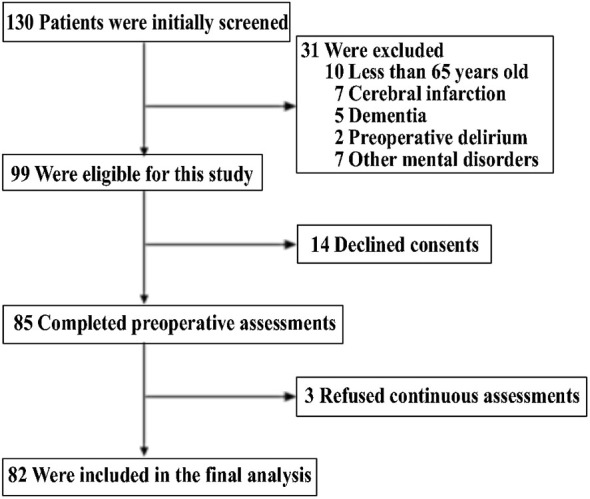
**Flow diagram**. The diagram shows that 130 participants were initially screened for the studies and 82 participants were included in the final data analysis.

One hundred and thirty patients were initially screened, and a total of 82 patients were included in the final data analysis (see Figure 1, the flow diagram). The demographic and clinical data of the participants are presented in Table 1. All of the participants had hip replacement (*N* = 43) or ORIF (*N* = 38) surgeries. Twenty-one of the 82 participants (25.6%) developed postoperative delirium either on day 1, day 2 or day 4. The prevalence of delirium in this patient population on day 1, day 2 and day 4 were 17 (20.7%), 17 (20.7%) and 13 (15.8%), respectively.

**Table 1:** **Demographic and clinical characteristics of the study population**.

Variables	Value
Age (years)
Mean ± SD	80.24 ± 6.00
Less than 75	15 (18.8%)
76–80	22 (27.5%)
81–85	32 (40.0%)
More than 86	11 (13.8%)
Gender, male (%)	18 (21.9%)
Marital status, married	78 (94.7%)
Height (cm) mean ± SD	155.00 ± 8.60
Weight (kg) mean ± SD	54.30 ± 9.13
BMI (kg/m^2^)	25.17 ± 3.25
Education (years) mean ± SD	4.20 ± 4.81
Disease, hip fracture	82 (100%)
Anesthesia, general anesthesia	82 (100%)
ASA class	
I	2 (2.6%)
II	51 (63.8%)
III	25 (31.3%)
Unknown	2 (2.5%)
Length of anesthesia (minutes) mean ± SD	127.09 ± 43.63
Length of operation (minutes) mean ± SD	91.66 ± 40.44
Estimated blood loss (mL) mean ± SD	314.49 ± 263.33
MMSE (points) mean ± SD	21.68 ± 5.28

*Abbreviation: ASA, American Society of Anesthesiologists; BDS, Blessed Dementia Scale; ADL, Activities of Daily Living; MMSE, Mini-Mental State Examination; SD, standard deviation; cm, centimeter; min, minute; kg, kilogram; mL, milliliter*.

### Reliability and validity of MDAS

The overall Cronbach’s alpha of the Chinese MDAS was 0.910. Table 2 shows the values of alpha for the MDAS in Chinese when a given item is removed. There was no single item that might affect the scale (alpha range between 0.899 and 0.913). The column “Item Total *r*” indicates the correlation between individual items and total MDAS scores. Defined by “Item-total *r*”, most items (1, 2, 3, 4, 5, 6, 7, 8 and 9) showed strong (*r* ≥ 0.7) or moderate (0.5 ≤ *r* < 0.7) correlation with the MDAS total score. Item 10 had a weak correlation with the total MDAS score (*r* < 0.5) (Table 2). These data point to the good internal consistency (reliability) of the MDAS.

**Table 2:** **Reliability and validity of Chinese version of MDAS**.

MDAS	Inter-item reliability	
Item	*α* if item removed	Item-total *r*
1	0.899	0.731**
2	0.905	0.858**
3	0.907	0.760**
4	0.907	0.769**
5	0.892	0.701**
6	0.892	0.760**
7	0.902	0.642**
8	0.903	0.580**
9	0.890	0.730**
10	0.913	0.343**
Total	—	0.910**

*Abbreviation: MDAS, Memorial Delirium Assessment Scale; CAM, confusion assessment method*.

*Note: Item-total and total-total *r*-values were based on Spearman rank-order correlation*.

**Correlation is significant at the 0.01 level (2-tailed).

Moreover, the participants who developed postoperative delirium had a higher MDAS score (average of day 1, 2 and 4 after surgery) than the participants who did not develop postoperative delirium: 11.44 ± 4.81 vs. 3.14 ± 1.81 (*P* < 0.0001, Student *t*-test).

### The optimal Chinese version MDAS cutoff point in describing postoperative delirium

**Figure 2:**
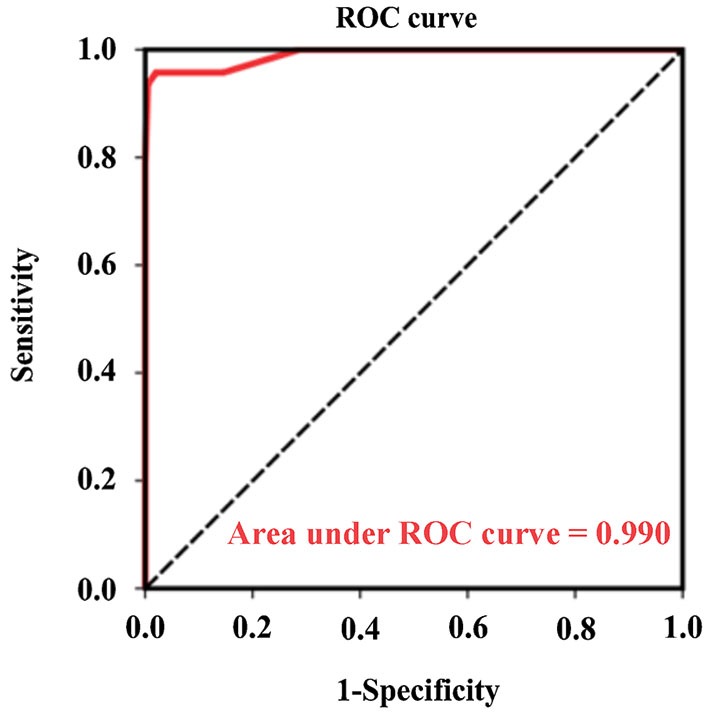
**Receiver operating characteristics (ROC) analysis was used for determination of the diagnostic sensitivity and specificity of the optimum value of the MDAS score vs. the CAM**. The area under the curve (AUC) is 0.990 (95% CI: 0.977–1.000, *P* < 0.001). Optimal cutoff point for MDAS is 7.5, at which point a sensitivity of 0.905 and a specificity of 0.984 are achieved. CI, confidence interval.

ROC analysis was performed to determine the optimal MDAS cutoff point, which combined the CAM-defined postoperative delirium assessments of days 1, 2 and 4. The area under the ROC curve was 0.990 (95% CI 0.977-1.000, *P* < 0.001) (Figure 2). An optimal MDAS cutoff point of 7.5, (combining the CAM-defined postoperative delirium assessments of days 1, 2 and 4), was obtained by Youden index (maximum of [sensitivity + specificity − 1]). Employing this defined cutoff point for the MDAS score, 45 out of 47 patients were identified as having delirium (defined by the CAM from days 1, 2 and 4), and two other patients were identified as not having delirium. The sensitivity of these identifications was 95.7%. Among the 199 patients without delirium (defined by the CAM from days 1, 2 and 4), 195 of them were identified as not having delirium and only four were identified as having delirium using this MDAS value (7.5). The specificity of the identification was 98.0%. Total identification accuracy of the MDAS vs. the CAM was 97.6%, and the Kappa value for concordance between the MDAS (using the cutoff value of 7.5) and the CAM, was 0.922 (Kappa statistic, *P* < 0.001). The positive and negative predictive values of this MDAS score (7.5) were 0.918 and 0.990, respectively (Table 3).

**Table 3:** **The sensitivity and specificity of optimal MDAS score in describing the CAM-defined postoperative delirium**.

Tests		CAM		Positive/Negative predictive values	Sensitivity/Specificity
		Delirium	Non-delirium
	Positive	45 (18%)	4 (2%)
MDAS				0.918/0.990	0.957/0.980
	Negative	2 (1%)	195 (79%)

*The optimal MDAS score was obtained by merging MDAS scores of postoperative days 1, 2 and 4. Among 47 patients, who were diagnosed with delirium according to the CAM, 45 of them were defined as having delirium by the MDAS using the cutoff score of 7.5. The positive predictive value is 0.918, the diagnostic sensitivity of the MDAS is 0.957, and the specificity is 0.980*.

## Discussion

In this prospective clinical study, we aimed to validate the MDAS with a Chinese population, and to determine the optimal MDAS cutoff point in identifying delirium with 82 Chinese patients who had hip surgery under general anesthesia. First, we found that the Chinese version of the MDAS has good reliability and validity. The data suggest that MDAS can be used in a Chinese population. Additionally, we found that a Chinese version MDAS score of 7.5, averaged from postoperative day 1, 2 and 4 scores, could be an optimal value for describing CAM-defined postoperative delirium in the patients who had hip surgeries under general anesthesia.

Previous studies have reported the prevalence of postoperative delirium in patients who had hip surgery for the repair of hip fractures as varying between 22.2% and 62.0% (Marcantonio et al., 2000, 2001; Gruber-Baldini et al., 2013; Bellelli et al., 2014; Brown et al., 2014; Holly et al., 2014). The postoperative delirium prevalence in the current study was 25.6% for the patients who had hip surgery, a similar value to one obtained in another study (Brown et al., 2014). The current study aimed to test the usefulness of the MDAS in postoperative delirium studies. We were able to find a significant difference in the MDAS scores of patients with postoperative delirium and patients without postoperative delirium, thus highlighting the effectiveness of the MDAS in identifying the presence and severity of delirium cases. Furthermore, the establishment of the current system will enable us to use the Chinese version of the MDAS to further assess the severity of delirium in the Chinese population in the future.

The MDAS has demonstrated good reliability and validity in clinical applications, and has retained its psychometric characteristics in different languages (Grassi et al., 2001; Matsuoka et al., 2001; Shyamsundar et al., 2009; Noguera et al., 2014). The current findings show that the MDAS in the Chinese language also had good internal consistency. Additionally, the MDAS in Chinese demonstrated a high degree of concurrent validity compared with the CAM (Table 3). As a result, these findings have established a system which will allow a larger scale study using both the CAM and the MDAS to be carried out in a future Chinese population.

The MDAS has been suggested to not only assess the severity of symptoms of delirium, but also to identify delirium, in previous studies. Specifically, Breitbart et al. reported that an MDAS score of 13 was an optimal value in identifying postoperative delirium in acquired immunodeficiency syndrome (AIDS) patients (Breitbart et al., 1997). Lawler et al. suggested an optimal MDAS score of 7, in another MDAS validation study, for cancer patients (Lawlor et al., 2000). Similarly, in the current study, an optimal MDAS cutoff point of 7.5 was obtained by ROC analysis based on the combined prevalence of delirium on postoperative day 1, day 2 and day 4. The optimal MDAS cutoff point of 7.5 identified most of the delirium patients determined by the CAM. These findings suggest that the Chinese version MDAS cutoff point of 7.5 offers optimal potential for determining the presence or absence of delirium; specifically, patients who had a MDAS score of 7.5, likely had delirium after hip surgery under general anesthesia.

The ROC result was close to ideal for the current study. These ideal findings are likely due to the fact that dementia patients were excluded from the cohort. The inclusion of participants with dementia would have led to less ideal ROC results, because participants with dementia would likely get higher MDAS scores even without delirium (Marcantonio et al., 2002).

We used the MMSE score of 18 as the cutoff value in defining cognitive impairment in the current study, as suggested in a previous study in a Chinese population (Katzman et al., 1998). The Chinese version of the MMSE, which includes five aspects (orientation, short-term memory, attention and calculation, language, and visuospatial), has demonstrated good reliability and validity among older Chinese adults (Katzman et al., 1998). The cutoff MMSE score in defining cognitive impairment is relatively lower in the Chinese population due to educational and cultural differences, which have been reported in previous studies (Zhang et al., 1990; Katzman et al., 1998; Sahadevan et al., 2001). Note that this MMSE cutoff score in identifying cognitive impairment has been accepted and is often utilized in studies in the Chinese population (Li et al., 2003; Zhou et al., 2006).

There are 10 items in the MDAS (Breitbart et al., 1997). The tenth item is the sleep-wake cycle. Interestingly, the tenth item demonstrated a weak correlation with the total MDAS score in the current study (*r* < 0.5) (Table 2). These findings suggest that sleep disturbance is common in both the participants with delirium and the participants without delirium. Future studies may aim to determine whether the item of sleep-wake cycle could be removed from the MDAS.

There were several limitations in the current study. First, patients were assessed for delirium only on postoperative days 1, 2 and 4, but not on day 3 or on later days after the surgery (e.g., postoperative day 7). However, most cases of postoperative delirium occur in the first 2 days after surgery. In addition, we only included participants who had hip replacements or open reductions with internal fixation under general anesthesia for the repair of hip fractures in the current study. It is possible that patients who have different types of surgeries (e.g., cardiac surgery) may have different optimal MDAS scores for the purpose of identifying postoperative delirium. Future studies may need to include patients undergoing other kinds of surgeries.

In conclusion, the results from the current study show that the MDAS in the Chinese language could be an effective and reliable method for determining the severity of delirium symptoms in older Chinese adults. Moreover, a cutoff score of 7.5 was discovered to have a very strong agreement with the CAM algorithm and can therefore be used to diagnose delirium. The findings from this pilot study have established a system and have provided preliminary data for future, larger-scale research with a Chinese population on postoperative delirium determined by using both the CAM and the MDAS. With the establishment of this current system, we should be able to use the Chinese version of the MDAS in the future to assess the severity of postoperative delirium in different Chinese populations.

## Conflict of interest statement

The authors declare that the research was conducted in the absence of any commercial or financial relationships that could be construed as a potential conflict of interest.

## Abbreviations

MDAS: Memorial Delirium Assessment Scale
CAM: Confusion Assessment Method
MMSE: Mini Mental State Examination.
